# Quantifying SARS-CoV-2 nucleocapsid antigen in oropharyngeal swabs using single molecule array technology

**DOI:** 10.1038/s41598-021-99807-7

**Published:** 2021-10-13

**Authors:** Dorte Aa. Olsen, Claus L. Brasen, Søren Kahns, Jeppe B. Madsen, Helene Kierkegaard, Henry Christensen, Anders Jensen, Thomas V. Sydenham, Jens K. Møller, Jonna S. Madsen, Ivan Brandslund

**Affiliations:** 1 Department of Biochemistry and Immunology, Lillebaelt Hospital, University Hospital of Southern Denmark, Vejle, Denmark; 2 Department of Clinical Microbiology, Lillebaelt Hospital, University Hospital of Southern Denmark, Vejle, Denmark; 3 Department of Regional Health Research, University of Southern Denmark, Odense, Denmark

**Keywords:** Biochemistry, Microbiology

## Abstract

This study aimed to develop a highly sensitive SARS-CoV-2 nucleocapsid antigen assay using the single molecule array (Simoa) technology and compare it with real time RT-PCR as used in routine clinical practice with the ambition to achieve a comparative technical and clinical sensitivity. Samples were available from 148 SARS-CoV-2 real time RT-PCR positive and 73 SARS-CoV-2 real time RT-PCR negative oropharyngeal swabs. For determination of technical sensitivity SARS-CoV-2 virus culture material was used. The samples were treated with lysis buffer and analyzed using both an in-house and a pre-commercial SARS-CoV-2 nucleocapsid antigen assay on Simoa. Both nucleocapsid antigen assays have a technical sensitivity corresponding to around 100 SARS-CoV-2 RNA molecules/mL. Using a cut-off at 0.1 pg/mL the pre-commercial SARS-CoV-2 nucleocapsid antigen assay had a sensitivity of 96% (95% CI 91.4–98.5%) and specificity of 100% (95% CI 95.1–100%). In comparison the in-house nucleocapsid antigen assay had sensitivity of 95% (95% CI 89.3–98.1%) and a specificity of 100% (95% CI 95.1–100%) using a cut-off at 0.01 pg/mL. The two SARS-CoV-2 nucleocapsid antigen assays correlated with r = 0.91 (P < 0.0001). The in-house and the pre-commercial SARS-CoV-2 nucleocapsid antigen assay demonstrated technical and clinical sensitivity comparable to real-time RT-PCR methods for identifying SARS-CoV-2 infected patients and thus can be used clinically as well as serve as a reference method for antigen Point of Care Testing.

## Introduction

Severe acute respiratory syndrome coronavirus 2 (SARS-CoV-2), a novel coronavirus, emerged in December 2019 in Wuhan, China^1^, and rapidly spread globally as the severe coronavirus disease 2019 (COVID-19) pandemic. In Denmark, the first patients with COVID-19 were diagnosed in February 2020. A key aspect in limiting transmission of this virus is the possibility to ensure an early and accurate diagnosis of individuals infected with SARS-CoV-2 allowing appropriate quarantine procedures for those infected. The standard testing strategy, as recommended by the World Health Organization (WHO, Geneva, Switzerland) targets SARS-CoV-2 RNA using polymerase chain reaction (PCR)^2^. During the spring of 2020, Roche Diagnostics (Basel, Switzerland) as a major supplier of PCR assays had insufficiency in the deliveries of reagents and consumables. This necessitated the consideration to use several other commercial and in-house methodologies for SARS-CoV-2 testing. In addition, in many laboratories, the PCR test capacity is challenged because of the high workload, need for skillful operators, and expensive instrumentations. We therefore determined whether a SARS-CoV-2 antigen assay using our established and highly sensitive technology, the single molecule array (Simoa) system (Quanterix©, Lexington, MA, USA) could serve as an alternative to the conventional PCR test to detect SARS-CoV-2. This would require a clinical sensitivity for diagnostic purposes comparable to the PCR methodology. Considering the fact that the Simoa technology has been shown to be up to 1000-fold more sensitive than conventional immunoassays^3,4^ we determined in this study whether a Simoa-based antigen test could serve as an alternative to the PCR test to detect SARS-CoV-2.

To our knowledge, Quanterix did not plan to construct a SARS-CoV-2 antigen assay, so we decided to develop an in-house SARS-CoV-2 antigen assay for the Simoa platform. Antigen methods for identifying SARS-CoV-2 focus on detection of the viral spike protein^5,6^ or the nucleocapsid protein^7,8^. Our early experiments revealed that nucleoprotein assays seemed to provide a superior diagnostic sensitivity as compared to spike protein assays. Thus, this work is focused on detection of the nucleoprotein to diagnose individuals infected with SARS-CoV-2.

In autumn of 2020, the in-house SARS-CoV-2 antigen assay reach a comparable sensitivity as compared to the real time RT-PCR technology. Meanwhile, Quanterix also succeeded in constructing a SARS-CoV-2 nucleoprotein antigen assay. Thus, this paper provides a head-to-head evaluation of both of these antigen-based tests compared to the conventional PCR test serving as the reference test to diagnose SARS-CoV-2.

## Materials and methods

### Sample material

Left over material from oropharyngeal swabs obtained from the daily clinic at the Department of Clinical Microbiology were stored in a clinical biobank after anonymization and according to guidelines at the Department of Clinical Microbiology, Lillebaelt Hospital. The study was performed according to the Danish data protection laws and EU GDPR. According to Danish health legislation § 42 (https://www.retsinformation.dk/eli/lta/2019/903) no informed consent from patients are needed in such kind of quality and method developments performed on anonymized surplus diagnostic material with no clinical or personal data.

Oropharyngeal swabs from a mixed population of symptomatic and asymptomatic individuals were available from 148 real time RT-PCR positive samples and 73 real time RT-PCR negative samples.

The swabs were diluted twofold in lysis buffer (100 mM Tris–HCL, 800 mM NaCl, 1% BSA pH 9.0, 1% Triton X-100) and incubated for 15 min followed by a centrifugation step at 10,000*g* for 5 min before analyzing for SARS-CoV-2 nucleocapsid antigen. Assay quality controls at three different levels were prepared in-house using a SARS-CoV-2 culture. The assay quality controls were treated equally to the samples and included in each run to evaluate assay performance and calculate the analytical coefficient of variation (CV%).

The viral RNA copy number equivalence of the SARS-CoV-2 culture was determined using the Bio-Rad SARS-CoV-2 droplet digital PCR (ddPCR) kit (Biorad, Hercules, CA USA) and expressed as number of SARS-CoV-2 RNA molecules/mL. The kit includes a SARS-CoV-2 RNA certified reference standard with a stated concentration (Exact Diagnostics, Fort Worth, TX, USA).

### SARS-CoV-2 real time RT-PCR

Oropharyngeal swabs were collected in MBT-010 tubes with 3 mL virus transport medium (MANTACC, Shenzhen, China) and sent within two hours to the Department of Clinical Microbiology at Lillebaelt Hospital (Vejle, Denmark). RNA was immediately extracted from the samples. Samples were vigorously vortexed for 15 s and 180 µL of the sample was transferred to a 96-well deepwell plate. Total RNA was extracted using MGIEasy Nucleic Acid Extraction Kit (MGI/BGI, Shenzhen, China) on the MGISP-960 High-throughput automated sample preparation system (MGI, Shenzhen. China) according to the manufactures instructions. RNA was eluted in 30 µL RNAse free water. Nuclease-free water was used as the non-template control and cultured SARS-CoV-2 virus was used as positive control material and included the extraction process. RT-PCR was performed on MX3005P instrument from Agilent (Santa, Clara, CA, US) using The RealStar^®^ SARS-CoV-2 RT-PCR Kit 1.0 (Altona Diagnostics, Hamburg, Germany) according to the manufacturer’s protocol. A final reaction volume of 30 μL containing 10 μL of template was used. The following cycling conditions were applied: a cDNA synthesis step (20 min/55 °C), a hold step (2 min/95 °C), and subsequently 45 cycles of denaturation (15 s/95 °C), annealing (45 s/55 °C), and elongation (15 s/72 °C).

### SARS-CoV-2 nucleocapsid antigen assay (in-house)

The SARS-CoV-2 nucleocapsid antigen in-house assay was developed on the automated Simoa HD-1 Analyzer platform (Quanterix©, Billerica, MA, USA). The following antibodies were tested (40143-R001, 40143-R004, 40143-MM05, 40143-MM08 (Sino Biological, Beijing, China) to find the best performing antibody pair^9^. Capture antibodies were covalently attached by standard carbodiimide coupling chemistry to carboxylated paramagnetic beads (Quanterix) using 0.2 mg/mL of antibody and 0.3 mg/mL of EDAC (Thermo Fisher Scientific, Waltham, MA, USA). Detector antibodies were biotinylated with 160 molar of biotin (Thermo Fisher Scientific) to antibody. As calibrator, a recombinant protein (40588-V08B, Sino Biological) was used. Streptavidin-β-galactosidase (SβG), enzyme substrate resorufin-β-D-galactopyranoside (RGP), and all consumables including wash buffers, cuvettes, disposable tips, and discs were from Quanterix.

The antigen assay was developed as a 2-step assay. Before running the following reagents were prepared; 1/3 of antigen beads was mixed with 2/3 of helper beads (Quanterix) in bead diluent buffer (Quanterix) to a final concentration of 2.0 × 10^7^ beads/mL. The biotinylated detector antibody was diluted in sample/detector diluent (Quanterix) to a final concentrations of 6.0 µg/mL. The SβG was diluted in SβG diluent (Quanterix) to 200 pM. After loading the prepared reagents and consumables, eight calibrators were prepared in sample/detection diluent using a threefold dilution starting at 80 pg/mL. Together with lysed swab samples and assay quality controls they were loaded onto the instrument in a 96-well microtiter plate. The calibrators were analyzed in duplicates while the quality controls and swab samples were run in single determinations. In each run, both RT-PCR negative and positive samples were included.

The following steps were performed by the instrument. 25 µL of bead mixture was pipetted into a cuvette together with 152 µL of sample, assay control, or calibrator, and 20 µL of biotinylated detection antibody. After 35 min of incubation the beads were magnetically separated and washed. 100 µL of SβG was added to the cuvette and incubated for 5 min. The beads were then separated magnetically and washed following the addition of RGP substrate. The bead substrate mixture was then loaded on to the Simoa disc containing an array of 216,000 micro-wells and sealed with oil. The concentrations of SARS-CoV-2 nucleocapsid antigen in the unknown samples were interpolated from the calibrator curve obtained by four parameter logistic regression fitting. The limit of detection (LOD) was determined to be 0.02 pg/mL using 3 × standard deviations from the blank. The analytical variation was calculated from 6 runs across multiple days resulting in 20%, 12% and 16% at level 0.3 pg/mL, 1.3 pg/mL and 13 pg/mL, respectively.

### SARS-CoV-2 nucleocapsid antigen assay (Quanterix)

A pre-commercialized version of the SARS-CoV-2 nucleocapsid antigen assay was provided by Quanterix. The assay uses eight calibrators ranging from 0 to 200 pg/mL. In brief, the assay is a 2 step assay using 25 µL of bead mixture (antigen beads and helper beads), 100 µL of sample and 20 µL of biotinylated detection antibody. An incubation step is performed for 35 min and after washing the beads, 100 µL of SβG is added and incubated for 5 min. The concentrations of SARS-CoV-2 nucleocapsid antigen in the unknown samples are interpolated from the calibrator curve obtained by four parameter logistic regression fitting. The calibrators are analyzed in duplicates, and the quality controls and swab samples are run in single determinations. In each run both RT-PCR negative and positive samples are included. The limit of detection (LOD) was determined to 0.15 pg/mL using 3 × standard deviations from the blank. The analytical variation was calculated from 13 runs across multiple days resulting in 20%, 16% and 14% at level 0.7 pg/mL, 2.7 pg/mL and 26 pg/mL, respectively.

### Statistical methods

Statistical analysis, data management and graphic presentations were performed using GraphPad Prism V.9.0.0 for Windows, GraphPad Software (San Diego, CA, USA). All data was found to be non-normally distributed and hence non-parametric statistic was used. For correlation analyses Spearman’s ρ were used. Reported P-values were two-sided and P < 0.05 were considered statistically significant. An in-house program was used for creating Table 1 according to Gerhardt and Keller^10^.

**Table 1:** Sensitivities and specificities depending on different cut-offs. True positive (TP), false negative (FN), true negative (TN), false positive (FP), positive predictive value (PPV), negative predictive value (NPV), not a number (NaN).

Cut-off pg/mL	Sensitivity (%)	95% CI (%)	Specificity (%)	95% CI (%)	TP (n)	TN (n)	FP (n)	FN (n)	PPV (%)	NPV (%)
**In-house assay**										
0.0001	100	96.9–100	0	0–4.9	118	0	73	0	62	NaN
0.001	94.9	89.3–98.1	97.3	90.5–99.7	112	71	2	6	98	92
0.01	94.9	89.3–98.1	100	95.1–100	112	73	0	6	100	92
0.1	92.4	86–96.5	100	95.1–100	109	73	0	9	100	89
1	89.8	83–94.6	100	95.1–100	106	73	0	12	100	86
**Quanterix assay**										
0.0001	100	97.5–100	0	0–4.9	148	0	73	0	67	NaN
0.001	95.9	91.4–98.5	94.5	86.6–98.5	142	69	4	6	97	92
0.01	95.9	91.4–98.5	95.9	88.5–99.1	142	70	3	6	98	92
0.1	95.9	91.4–98.5	100	95.1–100	142	73	0	6	100	92
1	91.2	85.4–95.2	100	95.1–100	135	73	0	13	100	85

## Results

### Developing an in-house assay for measuring SARS-CoV-2 nucleocapsid antigen

**Figure 1:**
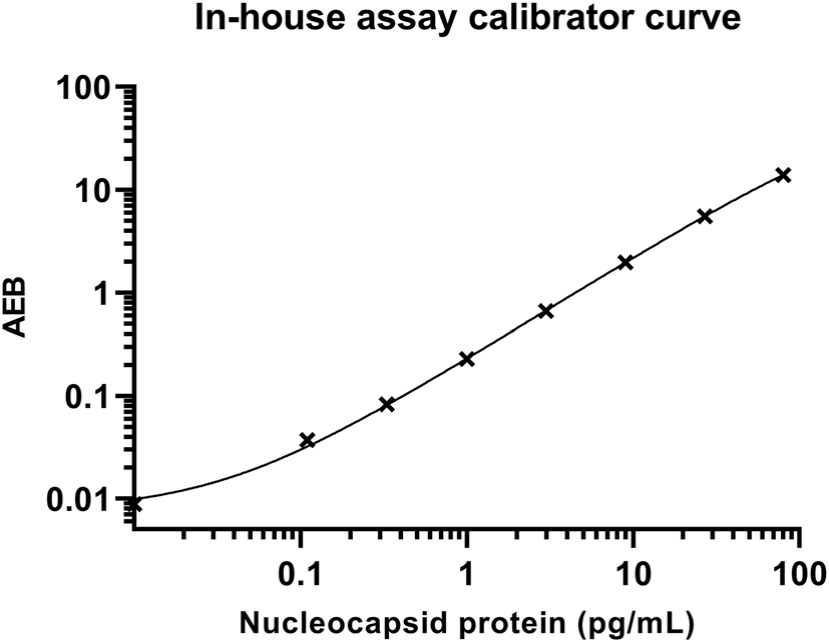
The calibrator curve of the in-house assay. The average number of enzyme per bead (AEB) against the concentration of nucleocapsid protein. Eight calibrators starting at 80 pg/mL and diluted using a threefold dilution. The calibrators were analyzed in duplicates and the mean values are depicted. The calibrator curve was a four parametric curve fit. The LOD was determined to be 0.02 pg/mL.

To achieve low background and a high signal to noise ratio for both calibrator and positive swab samples, various parameters were optimized. Initially, different antibody combinations were tested. The antibody pair (capture: 40143-MM08 and detection: 40143-R004) performed the best and was selected for further assay optimizations. Ratios of biotin to antibody for up to 320 molar excess were tested, detection antibody concentrations for up to 6.0 µg/mL and SβG concentration up to 200 pM, were also tested. Several sample diluents for preparation of the swab samples were examined as well as different running programs (2-step vs 3 step, volumes and minutes of incubation). The final parameters are described in material and methods. Figure 1 shows the calibrator curve of SARS-CoV-2 in-house assay.

### Determination of the technical sensitivity

**Figure 2:**
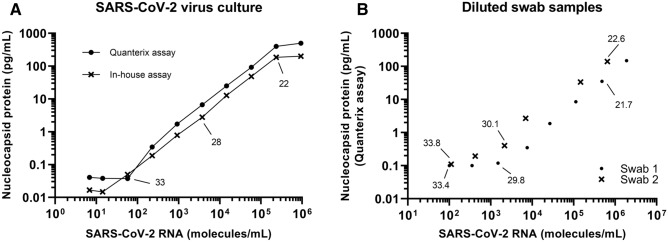
(**A**) A fourfold dilution of SARS-CoV-2 virus culture was analyzed using the Quanterix assay and the in-house assay. Nucleocapsid protein concentration against SARS-CoV-2 virus culture measured using ddPCR is shown. Real time RT-PCR Ct-values are presented. (**B**) Two real time RT-PCR SARS-CoV-2 positive swab samples were diluted using a fourfold dilution. Each dilution was analyzed for nucleocapsid protein using the Quanterix assay and for SARS-CoV-2 RNA using both real time RT-PCR and ddPCR. Real time RT-PCR Ct-values are shown.

The lower limit of detecting SARS-CoV-2 virus culture in the in-house assay and the Quanterix assay were both calculated using the LOD of the assays and estimated using Fig. 2A. A SARS-CoV-2 culture was diluted using a fourfold ranging from 3 to 8 × 10^5^ SARS-CoV-2 RNA molecules/mL. Every dilution was measured with both the in-house assay and the Quanterix assay (Fig. 2A). Using the LOD concentrations the in-house assay can detect a protein concentration corresponding to 20 RNA molecules/mL and the Quanterix assay 100 RNA molecules/mL. A technical sensitivity equivalent to 20–100 RNA molecules/mL is also found when estimating from Fig. 2A. The upper level of detection was 10^5^ RNA molecules/mL in both assays estimated from Fig. 2A. The technical sensitivity was further examined by preparing two real time RT-PCR positive swab samples in various dilutions and measuring using the Quanterix assay, ddPCR, and real time RT-PCR (Fig. 2B). Estimated from Fig. 2B the number of RNA molecules/mL were 2000 and 100 for swab 1 and 2, respectively. The upper level of detection was estimated to be 10^6^ RNA molecules/mL.

### Concentrations of SARS-CoV-2 nucleocapsid antigen in real time RT-PCR negative and positive samples

**Figure 3:**
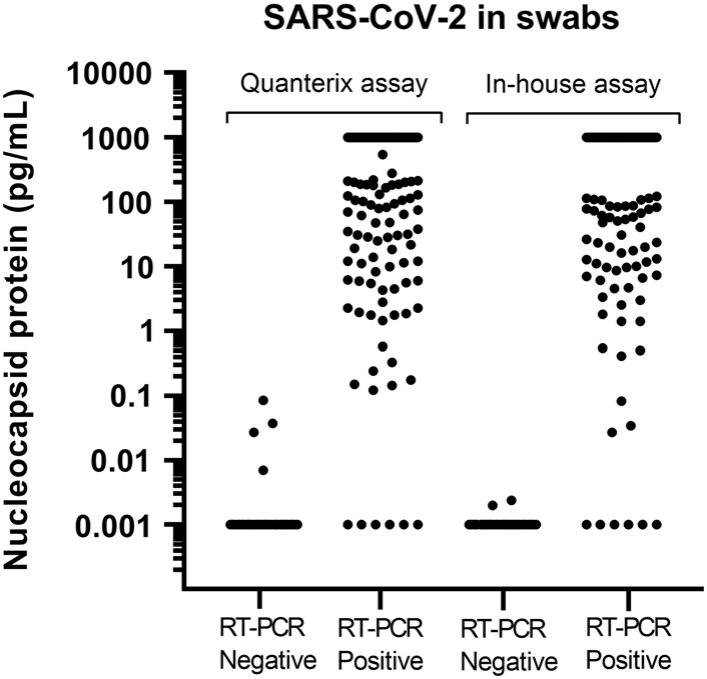
SARS-CoV-2 concentrations in real time RT-PCR negative and real time RT-PCR positive swab samples analyzed in the Quanterix assay and the in-house assay.

SARS-CoV-2 nucleocapsid antigen was measured in 73 real time RT-PCR negative samples and 148 real time RT-PCR positive samples using Quanterix assay (Fig. 3). Out of these samples there was enough material in 118 real time RT-PCR positive and 73 real time RT-PCR negative for quantifying SARS-CoV-2 nucleocapsid antigen using the in-house assay. The Ct-values of the positive samples were between 12.6 and 35.1 (median 20.5). For each sample, the signal coming from the helper beads was subtracted the concentration of the antigen beads which improved discriminating the positive from the negative samples. This procedure resulted in reported concentrations below assay LOD for a few samples. Samples with a concentration above the measuring range were given the concentration 1000 pg/mL and samples with concentrations below the calibrator curve were given the value 0.001 pg/mL.

In the in-house assay, the real time RT-PCR positive samples had antigen concentrations between 0.001 and 1000 pg/mL (median 111 pg/mL). 56 (47%) samples had concentration above the measuring range and 6 samples (5%) below. The antigen concentration in the real time RT-PCR negative samples were 0.001–0.0024 pg/mL (median 0.001 pg/mL), and 71 samples (97%) had concentrations below the measuring range. Applying a cut-off at 0.01 pg/mL resulted in 112 of the 118 real time RT-PCR positive samples were classified as antigen positive and 73 of the 73 real time RT-PCR negative samples were antigen negative (Table 1). This results in a sensitivity of 95% (95% CI 89.3–98.1%) and a specificity of 100% (95% CI 95.1–100%) (Table 1). The consequences of adjusting the cut-off or selecting a cut-off to obtain the best sensitivity or specificity are shown in Table 1.

In the Quanterix assay, the real time RT-PCR positive samples had antigen concentrations between 0.001 and 1000 pg/mL (median 215 pg/mL). Out of these, 71 (48%) samples had a concentration above the measuring range and 6 samples (4%) below. The antigen concentration in the real time RT-PCR negative samples were 0.001–0.085 pg/mL (median 0.001 pg/mL), and 69 samples (95%) had concentrations below the measuring range. Using a cut-off at 0.01 pg/mL resulted in 142 of 148 real time RT-PCR positive samples were found antigen positive and 70 of 73 real time RT-PCR negative samples were found antigen negative. This gives a sensitivity at 96% (95% CI 91.4–98.5%) and a specificity at 96% (95% CI 88.5–99.1%) (Table 1). Using a cut-off at 0.1 pg/mL resulted in a sensitivity at 96% (95% CI 91.4–98.5%) and a specificity at 100% (95% CI 95.1–100%) (Table 1). Five of the real time RT-PCR positive samples with Ct-values between 27 and 35 were found antigen negative in both antigen assays.

### Comparing the in-house assay and the Quanterix assay with real time RT-PCR Ct-values

**Figure 4:**
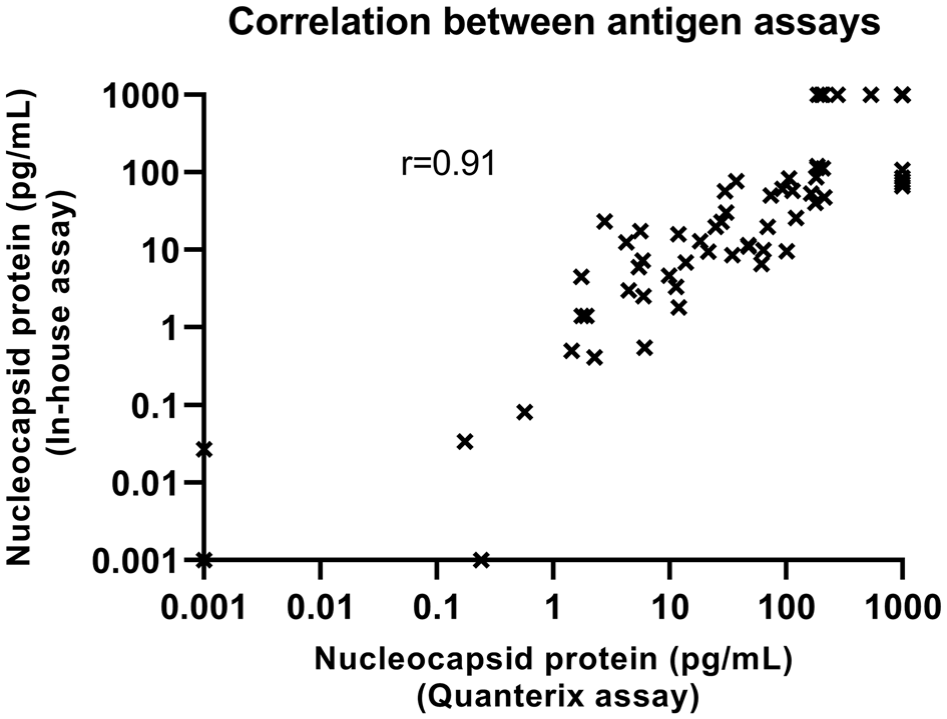
Correlation between the Quanterix assay and the in-house assay. The real time RT-PCR positive swab samples were used for the correlation. The r value calculated using Spearman’s ρ is shown.

**Figure 5:**
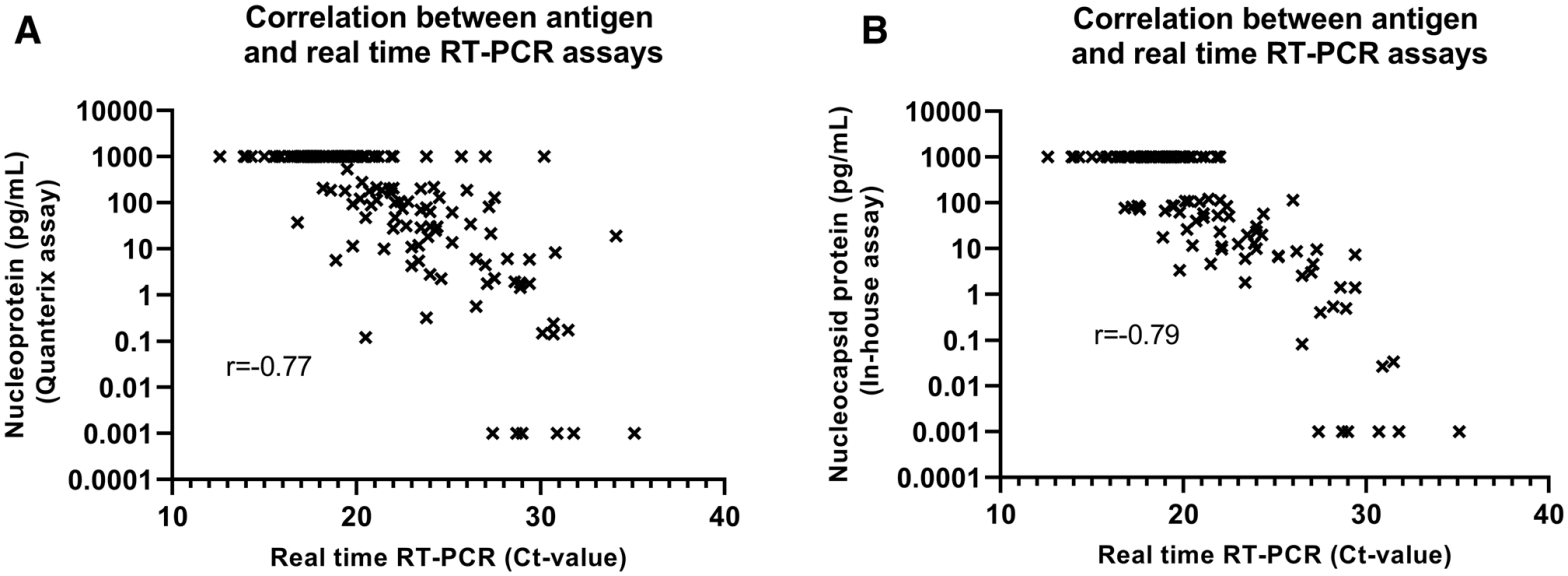
Correlation between nucleocapsid protein concentrations and real time RT-PCR Ct-values using the (**A**) Quanterix assay and the (**B**) in-house assay. The r values are presented.

The correlation between the in-house and the Quanterix assay was investigated using the SARS-CoV-2 antigen results of the real time RT-PCR positive samples (Fig. 4). The correlation was calculated to be r = 0.91, P < 0.0001. Moreover, correlations between antigen results and real time RT-PCR Ct-values were studied (Fig. 5A,B). Real time RT-PCR Ct-values correlated to antigen results using both the Quanterix assay (r = − 0.77, P < 0.0001) and the in-house assay (r = − 0.79, P < 0.0001).

## Discussion

In this study, an in-house assay was developed for measuring SARS-CoV-2 nucleocapsid antigen in oropharyngeal swabs from a mixed population of both symptomatic and asymptomatic individuals. The assay was compared with a pre-commercial SARS-CoV-2 nucleocapsid antigen assay from Quanterix, but FDA EUA approved on January 11, 2021. We demonstrated that the in-house assay and the Quanterix assay are highly correlated and can measure the nucleocapsid protein with similar technical sensitivity as determined by comparing to a virus culture with a measured concentration of SARS-CoV-2 RNA molecules. As the analytical coefficient of variation measured as a total day-to-day variation is the same for the two assays, between 12 and 20%, they seem equally acceptable for clinical use.

The question is, however, whether this technical sensitivity is sufficient for a clinical diagnostic purpose. The two antigen assays performed equal well and both assays are capable of discriminating real time RT-PCR positive samples from real time RT-PCR negative patients with sensitivities of 95–96% and specificities of 96–100% depending on the applied cut-off value. Five samples that were positive with real time RT-PCR (Ct-values 27–35) were negative in both nucleocapsid antigen assays. Unfortunately, it was not possible to repeat the real time RT-PCR measurements of these samples due to insufficient amounts of remaining sample material but the divergence could be explained by the real time RT-PCR methodologies having higher analytical sensitivity. Another explanation could be that we measure the nucleocapsid protein of the virus while the real time RT-PCR method measures the presence of RNA in the samples. Only one other study has quantitated SARS-CoV-2 nucleocapsid antigen in swabs with a sensitivity similar to the present methods^7^. This is understandable as standard immunoassays are less sensitive as compared with the Simoa technology^3,11,12^. Other studies measuring SARS-CoV-2 antigen with similar sensitivities have focused on measuring the nucleocapsid protein in blood^6,8,13^.

Real time RT-PCR is considered the gold standard for the diagnosis of SARS-CoV-2 infection. Antigen detection tests are more rapid than laboratory based PCR, less laborious, and less expensive but still require clinical validation. Recently rapid diagnostic tests have been introduced as screening in Denmark using the lateral flow system. Rapid antigen tests detect SARS-CoV-2 antigen in as little as 10–15 min and have shown very varying sensitivities. As compared to real time RT-PCR, Krüger et al. found sensitivities between 50 to 77% using three different antigen rapid tests^14^. In two different populations, Gremmels et al. found sensitivities of 73 and 81%^15^. In a review by Dinnes et al., an average sensitivity of 56% was found based on 5 studies^16^. A new study in Denmark has found a sensitivity of 70% including 4697 individuals^17^. The SARS-CoV-2 antigen assays described in this paper are highly sensitive and could thus be used as a reference method for antigen POCT. Furthermore, the assays are quantitative and an advantage is that the cut-off is not fixed and pre-determined as it is for POCT but can be adjusted to reach a desired sensitivity or specificity depending on the purpose of the assay.

Mutational changes in the gene coding the nucleocapsid antigen might change the antigenic structure to a degree to escape detection by the antibody. If this happens there is no problem in changing to a new relevant antibody, as the system is easily modified and again check the sensitivity and specificity. This might be difficult and time consuming for other methodologies.

The present SARS-CoV-2 antigen assays can produce within a total turn-around time of 3 h from sampling to reportable results, around 100 tests, yielding a minimum of 800 results per 24 h depending on staffing and organization. If 250 tests are done with a reportable test result time of 5 h the capacity could reach between 1000 and 1200 tests per 24 h. For the pre-commercial assay the price would be 21 dollars per reportable result including reagent and technologist costs. Though it would be less expensive to use the in-house SARS-CoV-2 assay, a commercial produced kit is preferable if to be used by other laboratories. This will ensure a certain standardization in the single measurements and from batch to batch when produced by a commercial company, which also includes control materials in the kit. However international reference materials both as RNA and protein with certified values are needed in future method comparisons.

We have also tested the capability of the method for measuring and quantitating the nucleocapsid protein in blood of patients. It seems that the protein can be reliably measured and patients followed during their disease process until discharged with no virus in the blood^18^.

In conclusion, we show that the in-house and the pre-commercial SARS-CoV-2 antigen assays are comparable and both can be used for discriminating SARS-CoV-2 positive oropharyngeal swabs from negative with sensitivities comparable to real time RT-PCR.

## Data Availability

Anonymous data are available from the corresponding author upon request.

## Abbreviations

Simoa: Single molecule array
SβG: Streptavidin-β-galactosidase
RGP: Resorufin-β-d-galactopyranoside
AEB: Average number of enzymes per bead
RT-PCR: Reverse-transcription polymerase chain reaction
POCT: Point Of Care Testing
